# Psychological indicators for healthy aging: validation of the German short version of Ryff’s scales of psychological well-being (SPWB)

**DOI:** 10.1186/s41687-025-00854-9

**Published:** 2025-02-25

**Authors:** Ana N. Tibubos, Anna C. Reinwarth, Iris Reiner, Antonia M. Werner, Philipp S. Wild, Thomas Münzel, Jochem König, Karl J. Lackner, Norbert Pfeiffer, Manfred. E. Beutel

**Affiliations:** 1 Department of Psychosomatic Medicine and Psychotherapy, University Medical Center of the Johannes Gutenberg-University Mainz, Mainz, Germany; 2 Diagnostics in Healthcare and E-Health, Trier University, Trier, Germany; 3 Department of Psychiatry and Psychotherapy, University Medical Center of the Johannes Gutenberg-University Mainz, Mainz, Germany; 4 Preventive Cardiology and Preventive Medicine-Department of Cardiology, University Medical Center of the Johannes Gutenberg-University Mainz, Mainz, Germany; 5 Center for Thrombosis and Hemostasis (CTH), University Medical Center of the Johannes Gutenberg-University Mainz, Mainz, Germany; 6 German Center for Cardiovascular Research (DZHK), Partner Site Rhine-Main, Mainz, Germany; 7 Institute of Molecular Biology (IMB), Mainz, Germany; 8 Department of Cardiology – Cardiology I, University Medical Center of the Johannes Gutenberg-University Mainz, Mainz, Germany; 9 Institute of Medical Biostatistics, Epidemiology and Informatics (IMBEI), University Medical Center of the Johannes Gutenberg-University Mainz, Mainz, Germany; 10 Institute of Clinical Chemistry and Laboratory Medicine, University Medical Center of the Johannes Gutenberg-University Mainz, Mainz, Germany; 11 Department of Ophthalmology, University Medical Center of the Johannes Gutenberg-University Mainz, Mainz, Germany

**Keywords:** Eudaemonic well-being, Scales of Psychological Well-being (SPWB), Ryff scales, Positive psychology, Healthy aging, Psychometric properties

## Abstract

**Background:**

The important roles of well-being as realization of one’s true potential for healthy aging have been highlighted by literature of the recent decades. The Scales of Psychological Well-being (SPWB) are an internationally recognized measurement tool for psychological well-being. Yet, sound validation of the German SPWB 18-item version has been lacking to date. Therefore, the present study aims to (1) test the psychometric properties of the German SPWB 18-item version in terms of factorial validity and (2) determine construct validity by investigating its correlations with psychosocial variables, mental and physical health. (3) Sociodemographic characteristics of the SPWB in a middle to old age German population was explored.

**Methodology:**

Data of *N* = 3,374 participants 45–85 years old of the 10-year follow-up (2017–2022) of the Gutenberg Health Study (GHS) were analyzed. Descriptive analyses and inference statistical analyses were performed to assess construct validity. In order to determine the psychometric properties, item characteristics and reliability coefficients were analyzed. Confirmatory factor analyses tested the proposed theoretical factorial structure.

**Results:**

Construct validity of the SPWB was established with respect to sociodemographic, psychosocial (social support and resilient coping), and health variables (sleeping problems, depression and anxiety symptoms, stress, loneliness, and somatic diseases). Analysis of the psychometric properties of the German SPWB 18-item version rendered support for the theoretically proposed multidimensional structure of psychological well-being in our sample rather than a one factorial structure. Bi-factor models that take the method effects of positively and negatively formulated items into account are highly recommended.

**Conclusions:**

The German SPWB 18-item version shows comparable psychometric properties to previous large-scale studies from other countries. The SPWB provides psychological indicators for healthy aging.

## Introduction

The importance of well-being for maintaining physical and mental health has been increasingly highlighted in ageing [1]. The World Health Organization (WHO) has defined healthy ageing as “… the process of developing and maintaining the functional ability that enables well-being in older age. Functional ability reflects a person’s physical and mental capacities, the environments he or she inhabits and the ways in which people interact with their environment” [2].

In an influential concept, Diener [3] has defined subjective well-being as the frequent occurrence of positive emotions in connection with a less frequent occurrence of negative emotions and a positive assessment of life satisfaction, emphasizing the hedonic components. Psychological well-being in terms of eudaemonic well-being, in contrast, focuses on positive human functioning which is considered emotion-independent [4, 5]. A theoretical model and internationally recognized measurement tool of psychological well-being was first established by Ryff in 1989 [4, 6]. His *Scales of Psychological Well-being (SPWB)* contain six subscales which originate in Aristotle’s Nicomachean Ethics while drawing content from various developmental, humanistic, and psychoanalytic theories [4]. Ryff assumes that well-being in life is due to the fulfillment of a series of developmental tasks. Thus, well-being is both a state and an active coping with challenges conditioned by one’s personality traits and inherent action tendencies, society, and culture [7]. The multidimensional and resource oriented concept of psychological well-being focusses on individual potential and not on inevitable age-related deficits and diseases, providing a measurement approach to healthy aging in accordance with the current global strategy of the WHO [8].

The dimension of *Self-acceptance* refers to appreciation of both positive and negative characteristics of one’s self [9]. *Positive relations with others* are characterized by trust and intimacy. *Purpose in life* describes the existence of plans and goals oriented toward higher, meaningful values. Ryff defines *Autonomy* as the ability to think, assess and act autonomously, without being influenced by external opinions, while *Personal growth* represent the tendency to develop continuously along one’s own values. *Environmental mastery* refers to the ability to influence one’s own environment.

To date, evidence regarding the association of SPWB and sociodemographic characteristics has been limited. In one of the few longitudinal studies, little age-associated variation from middle (> 32 years) to old age (up to 75 years) was found [10]. *Personal growth* and *Purpose in life* were the two dimensions with consistent decline in higher age. In Lee et al. [11], older adults also reported lower SPWB compared to younger adults, especially regarding *Purpose in life* and *Personal growth*. Regarding gender, in a Spanish study [12], elderly men scored higher regarding *Self-acceptance*, *Autonomy*, *Purpose of life* and *Environmental mastery* compared to elderly women.

From an aging perspective, the subscale *Purpose in life* has been getting considerable attention. Based on US data of the Health and Retirement Study, Alimujiang et al. [13] found that participants with the highest (vs. lowest) *Sense of purpose* had a considerably lower mortality. Participants with the highest sense of purpose also reported fewer sleeping problems, less loneliness, more optimism and a lower risk of depression four years later [14]. Other studies emphasized the relevance of *Self-acceptance*. For instance, a positive link between self-acceptance and mental health in terms of somatization, obsessive-compulsive, interpersonal sensitivity, depression, anxiety, hostility, phobic anxiety, paranoid ideation, psychoticism, and psychological distress was established at cross-sectional level and four years later in the German MainLife - Longitudinal Study of Brief Entire Life Narratives [15]. In a recent study, network analysis of the SPWB 29-item version in a Spanish sample suggested that *Self-acceptance* is the most central dimension in Ryff’s SPWB [16]. Further, psychological well-being was positively associated with self-awareness, outlook/attitude, life-long learning, social support, and faith [1], psychological resilience and competence [17], and negatively associated with depression and general psychopathology [18].

Despite its widespread use in research as a good indicator of psychological well-being, the theoretical six-factor model of SPWB has still remained an issue of discussion. For the short form with 18 items, Ryff and Keyes found a multidimensional model with six first-order factors and one single second-order factor of psychological well-being as best fitting a nationally representative U.S. sample of adults above the age at 25 years [5]. For further overview of psychometric analyses of the short form of SPWB, see [19] and [20]. In psychology student samples in the Netherlands, the psychometric quality of the SPWB was tested for three different versions with 3, 9, and 14 items per scale [21]. However, the validity of the proposed theoretical six-factor model was only acceptable for the 3-items per scale version. To our knowledge, with regard to the development of the German version of this short form [22], no published study on the psychometric properties is available to date.

## Objective of the study

Given the significance of psychological well-being for healthy aging, we aim to investigate the psychometric properties and validity of the German SPWB 18-item version by Staudinger et al. [22]. First, we test the psychometric properties (item characteristics, reliability, proposed theoretical factorial structure) of the German SPWB 18-item version in a population-based German cohort-study covering the adult life span from middle to old age. Second, we determine construct validity by analyzing SPWB and its associations with psychosocial variables (resilience, social support, loneliness), mental and physical health (number of reported somatic diseases, sleep problems, depression, anxiety, stress). Based on previous work, negative associations with number of reported sleeping problems, depression, anxiety, stress, loneliness, and somatic diseases are expected on the one hand. On the other hand, positive associations regarding resilience and social support are expected. Third, we explored its sociodemographic characteristics regarding to age, sex, marital status, education, occupation, and income in a middle to old age German population.

## Methods

### Sample & data collection

Data of the Gutenberg Health Study (GHS) was used, an ongoing population-based, prospective, single-center cohort study in the Rhine-Main region located in western Mid-Germany started in 2007 [23]. Its primary aim is to analyze and improve cardiovascular risk factors and their stratification.

The sample was drawn randomly from the local registries of the city of Mainz and the district of Mainz-Bingen, stratified 1:1 for gender and residence (city vs. district) and in equal strata across age decades. Eligibility criteria was age 35 to 74 at baseline with 15,010 initial participants. Insufficient knowledge of the German language, and physical or mental inability to visit the study center for study investigations were exclusion criteria. During an extensive 5-hour examination in the study center, cardiovascular risk factors and other clinical variables were assessed, complemented by a computer-assisted personal interview, laboratory examinations from venous blood samples, blood pressure, and anthropometric measurements. All examinations were performed by certified medical technical assistants following standard operating procedures.

The present study is based on the 10-year follow-up examination between 2017 and 2022 in which the SPWB was administered for the first time. For this follow-up, 10,000 participants of the baseline cohort were addressed. The cleansed dataset regarding SPWB consisted of *N* = 3,374 participants with 1,626 men (48%) and 1,748 women (52%) with a mean age of 60,98 years (SD = 13.74).

### Variables and measures

The German SPWB [5, German version: 22] 18-item version assessed psychological well-being. The SPWB assesses six different facets of psychological well-being: *Autonomy*, *Personal growth*, *Environmental mastery*, *Purpose in life*, *Positive relations with others* and *Self-acceptance*. Each item is rated on a six-point scale (1 = *strongly disagree* and 6 = *strongly agree*). Eight negatively formulated items were reversed before scale aggregation for a total score and subscale scores. High values represent a high level of psychological well-being. Detailed psychometric properties are reported in the results section.

Sociodemographic characteristics were assessed via self-report: gender (1 = male, 2 = female), age (years: 1 = 45–54, 2 = 55–64, 3 = 65–74, 4 = > 75), partnership (0 = no, 1 = yes), education (1 = school-leaving qualification after 9 years, 2 = school-leaving qualification after 10 years, 3 = technical college certificate or higher education entrance qualification, 4 = other or none), and employment (1 = full-time, 2 = part-time, 3 = irregular, 4 = retired, 5 = unemployed). Equivalence income was calculated by integrating the two variables household income (25 categories overall, starting with < 150€, 150–399€, 400–499€, 500–749€ and up to > 20,000€) and number of individuals in household [24].

To establish construct validity, measures of mental health that have previously been found to be significantly associated with psychological well-being were assessed, including depression, sleep disorder, loneliness, psychological distress, resilience and social support. Depressive symptoms were measured with the nine items of the depression module of the Patient Health Questionnaire (PHQ-9) [25]. In the present sample, the PHQ-9 showed good internal consistency (ω = 0.85). Generalized anxiety was assessed with the 7-items short form of the Generalized Anxiety Disorder (GAD-7) [26]. In the present sample, reliability of GAD-7 is excellent (ω = 0.89). Using a Likert scale ranging from 0 = not at all to 3 = nearly every day, participants are asked to indicate how often they were bothered by the respective symptom of depression or anxiety over the course of the last two weeks.

The German version of the Jenkins Sleep Survey JSS-4 [27] was used to assess subjective sleep quality in terms of sleep problems. The response format of the 4-item questionnaire with excellent reliability in the present sample (ω = 0.89) is a 6-point scale (0 = never to 5 = 22–31 days) refers to the past four weeks.

Loneliness was assessed using one validated item: ’I am frequently alone /have few contacts’ rated from 0 = no, does not apply, to 4 = yes, it applies, and I suffer strongly from it [28, 29].

The PHQ Stress module assesses psychosocial strain during the last month by ten items with acceptable reliability (ω = 0.82) [30]. It includes health, work/financial, social and traumatic stress. Its response options range from 0 = not bothered at all to 2 = bothered a lot.

The Brief Resilience Coping Scale (BRCS) [31, 32] captures resilient coping style reliably (ω = 0.80) based on four items with a 5-point Likert scale (1 = not at all to 5 = very).

Social support was measured using the Brief Social Support Scale (BS6) [33] with six items on emotional-informational and tangible social support. Respondents indicated on a 4-point scale how often such support was available for them. The response options were “always” (1), “mostly” (2), “sometimes” (3), and “never” (4). In the present sample, internal consistency of the total scale was good (ω = 0.82).

As part of a computer-assisted personal interview, participants were asked whether they had ever received a definite diagnosis of specific physical disorders by a physician: cardiovascular disease (e.g. hypertension, coronary heart disease, cerebrovascular disease, peripheral artery disease, congestive heart failure, atrial fibrillation), cancer, migraine and pulmonary diseases (asthma, chronic obstructive pulmonary disease), hypertension and diabetes).

### Statistical analyses

For data preparation and analyses SPSS (version 28.0.1.1) and R (4.1.2) were used. Mean scores were calculated for the scale aggregation.

Descriptive statistics including thorough item- and scales statistics and inference statistical analyses (ANOVA: Analysis of Variance, Spearman correlations) were performed. For reliability analysis, Cronbach’s alpha was calculated as a measure of internal consistency for each scale. Effect sizes for ANOVAs η² represent small ≥ 0.010, medium ≥ 0.060, and large ≥ 0.140 effects [34]. To test construct validity associations of SPWB, sociodemographic characteristics were analyzed and intercorrelations with outcomes regarding psychological variables related to SPWB, physical and mental health were investigated.

Different factor structures (1-factor, correlated 6-factor, second-order factor model and corresponding bi-factor models taking possible methods effect into account due to positively and negatively formulated items) were tested on item level: (1) 1-factor model with only a general factor for psychological well-being, (2) correlated 6-factor model representing the six postulated subscales of psychological well-being, (3) second-order factor model with a general factor for psychological well-being with six first-order factors representing the six subscales. Model 2) and 3) represent the proposed multidimensional model of psychological well-being proposed by Ryff. Additionally, corresponding bi-factor models taking positively and negatively formulated items from a methodological perspective into account were tested: 3) bi-factor model based on 1-factor model with only a general factor for psychological well-being, 2) bi-factor model based on correlated 6-factor model representing the six postulated subscales of psychological well-being, 3) bi-factor model based on second-order factor model with a general factor for psychological well-being with six first-order factors representing the six subscales. Factor structure was tested by running confirmatory factor analyses (CFA) using the maximum likelihood with robust standard errors (MLR) since the SPWB items with a 6-point rating scale were almost normally distributed and our analysis sample was fairly large. For scaling purposes, a value of one is assigned to one of the factor loadings. Model fit was estimated using primary fit indices as recommended by Hu, Bentler [35]: chi-square test of model fit (χ^2^), comparative fit index (CFI), root mean square error of approximation (RMSEA) including the 90% confidence intervals, and standardized root mean square residuals (SRMR). The χ^2^-test should ideally not be significant or χ^2^ divided by degrees of freedom (χ^2^/df) should be smaller than 3 for an excellent model fit. For the CFI, a value close to 1 exemplifies an excellent model fit, a value > 0.95/0.90 a very good/acceptable model fit. For the SRMR and RMSEA, a value close to 0 denotes a perfect model fit, whereas values 0.06/0.08 are good/acceptable.

## Results

### Sociodemographic characteristics

Descriptive statistics of the SPWB total score and the theoretically proposed six subscales are presented in Table 1 with regard to sociodemographic variables.

No significant gender difference was observed, except for women scoring higher on the subscale *Positive relations to others* with a small effect size.

Analyses stratified for age revealed that psychological well-being, both at subscale and total score level, was consistently lowest in the age category 75 years and older, except for *Environmental mastery*. *Personal growth* and especially *Purpose in life* significantly decreased continuously, with even moderate effect size for the latter. The highest scores were found for the age group from 65 to 74, regarding total score, *Autonomy*, *Environmental mastery*, and *Self-acceptance*. No significant age-related results were found for *Positive relations to others*.

Regarding partnership, significant differences with small effect sizes were observed except for *Autonomy*. Individuals with a partner tended to have higher scores on psychological well-being in general as well as in its several subdimensions.

Psychological well-being significantly depended on the educational background, with a small effect size at total score level. Individuals with higher education reported the highest scores on all facets of psychological well-being. The difference between those with low and high education was largest for *Personal growth*, with almost moderate effect size. No significant differences were found for the subscales *Environmental mastery*, *Positive relations to others*, and *Self-acceptance*.

Employment status was weakly associated with psychological well-being in general. At subscale level regarding *Personal growth* and even more *Purpose in life*, individuals with a full time or part-time job reported significantly higher scores compared to individuals without jobs. Retired people reported the highest score in *Environmental Mastery* compared to employed or unemployed.

Spearman correlation analyses of equivalence income and SPWB revealed weak positive correlations (range *r* =.06 −.21). General psychological well-being and the subscale *Personal growth* showed the highest correlations, while *Autonomy* and *Positive relations to others* showed the lowest. Details are displayed in Table 2.

### Validity based on health and psychosocial variables

All correlation coefficients are reported in Table 2. Correlational analyses with external criteria underscore the construct validity of the Ryff scales. As expected, negative relationships were observed between the Ryff subscales and total score with number of reported sleeping problems (JSS-4), depression (PHQ-9), anxiety (GAD-7), stress (PHQ-Stress), loneliness, and somatic diseases on the one hand. On the other hand, positive correlations were observed regarding resilient coping style (BRCS) and perceived social support (BS6). Overall, psychological well-being was moderately associated with almost all health-related and psychosocial variables. Weaker associations were observed for sleeping problems and number of somatic diseases. At subscale level, especially *Environmental mastery* and *Self-acceptance* were strongly associated with the analyzed external criteria, in particular with psychological health. *Personal growth* had the strongest correlation with resilient coping style. *Positive relations to others* turned out to show the strongest correlation with loneliness, followed by *Environmental mastery* and *Self-acceptance.* No significant associations were found between sleeping problems and the subscales *Autonomy* and *Purpose in life* nor between stress and *Self-acceptance*. Also, the number of somatic diseases did not correlate with *Autonomy*, *Positive relations to others*, and *Self-acceptance.* Although with small effect size, higher SPWB is more likely if there is no somatic disease (see Table 1).

**Table 1:** Descriptive and inference statistics of SPWB regarding sociodemographic and health condition (*N*_total_ = 3,374)

Variable (*n*)	SPWB total score		Autonomy		Environmental mastery		Personal growth		Positive relations to others		Purpose in life		Self-acceptance	
	M (SD)	η²	M (SD)	η²	M (SD)	η²	M (SD)	η²	M (SD)	η²	M (SD)	η²	M (SD)	η²
Sex		0.001		0.001		0.001		0.000		0.016***	3	0.000		0.001
*Male (1*,*748)*	3.57 (0.58)		3.30 (0.88)		3.84 (0.89)		3.78 (0.91)		3.23 (1.00)		3.46 (0.93)		3.77 (0.97)	
*Female (1*,*626)*	3.61 (0.61)		3.25 (0.94)		3.80 (0.93)		3.77 (0.97)		3.49 (1.02)		3.49 (0.94)		3.83 (1.02)	
Age (years)	3.58 (0.60)	0.012***	3.27 (0.91)	0.011***	3.82 (0.91)	0.010***	3.77 (0.94)	0.030***	3.36 (1.02)	0.003*	3.47 (0.94)	0.061***	3.80 (0.99)	0.003*
*45–54 (708)*	3.62 (0.57)		3.10 (0.91)		3.73 (0.89)		3.94 (0.82)		3.44 (1.05)		3.75 (0.82)		3.77 (1.00)	
*55–64 (981)*	3.61 (0.59)		3.29 (0.87)		3.76 (0.91)		3.89 (0.90)		3.32 (1.00)		3.63 (0.86)		3.80 (0.96)	
*65–74 (881)*	3.63 (0.57)		3.37 (0.85)		3.96 (0.83)		3.75 (0.90)		3.39 (0.98)		3.40 (0.93)		3.88 (0.92)	
*75+ (804)*	3.47 (0.64)		3.31 (1.01)		3.85 (0.98)		3.51 (1.05)		3.29 (1.05)		3.12 (1.00)		3.74 (1.08)	
Partnership		0.012***		0.000		0.009***		0.003**		0.008***		0.005***		0.011***
*no (819)*	3.48 (0.63)		3.28 (0.92)		3.69 (0.97)		3.71 (0.98)		3.20 (1.04)		3.38 (0.95)		3.63 (1.07)	
*yes (2*,*440)*	3.63 (0.58)		3.28 (0.91)		3.88 (0.88)		3.82 (0.91)		3.42 (1.00)		3.53 (0.92)		3.87 (0.95)	
Education		0.016***		0.003*		0.001		0.057***		0.002		0.033***		0.001
*1 (1*,*139)*	3.51 (0.63)		3.23 (0.98)		3.81 (0.96)		3.53 (0.98)		3.33 (1.04)		3.25 (0.98)		3.84 (1.01)	
*2 (855)*	3.58 (0.58)		3.27 (0.88)		3.82 (0.88)		3.72 (0.93)		3.37 (0.98)		3.53 (0.89)		3.76 (1.01)	
*3 (1*,*346)*	3.67 (0.57)		3.23 (0.87)		3.85 (0.87)		4.03 (0.84)		3.38 (1.03)		3.64 (0.88)		3.79 (0.96)	
*4 (23)*	3.72 (0.47)		2.96 (0.84)		3.57 (1.12)		3.29 (1.05)		2.86 (0.98)		3.12 (0.87)		3.85 (0.79)	
Employment		0.007***		0.003		0.005**		0.027***		0.008***		0.049***		0.002
*Full-time (1*,*061)*	3.63 (0.56)		3.22 (0.86)		3.79 (0.86)		3.95 (0.85)		3.29 (1.03)		3.71 (0.84)		3.82 (0.97)	
*Part-time (365)*	3.69 (0.57)		3.25 (0.97)		3.78 (0.92)		3.94 (0.88)		3.59 (0.93)		3.72 (0.84)		3.86 (0.93)	
*Irregular (177)*	3.56 (0.61)		3.23 (0.91)		3.77 (0.94)		3.80 (0.92)		3.40 (1.02)		3.44 (0.96)		3.73 (1.01)	
*Retired (1*,*469)*	3.55 (0.61)		3.32 (0.93)		3.90 (0.92)		3.63 (0.97)		3.35 (1.01)		3.28 (0.97)		3.81 (0.99)	
*Unemployed (162)*	3.58 (0.58)		3.25 (0.92)		3.69 (0.94)		3.86 (0.84)		3.45 (1.01)		3.57 (0.79)		3.65 (1.04)	
Somatic diseases		0.007***		0.000		0.002**		0.015***		0.001		0.009***		0.000
*0 (2*,*089)*	3.62 (0.57)		3.27 (0.90)		3.86 (0.87)		3.86 (0.89)		3.38 (1.01)		3.54 (0.91)		3.82 (0.97)	
*1+ (1*,*276)*	3.52 (0.63)		3.28 (0.94)		3.77 (0.97)		3.63 (0.99)		3.31 (1.02)		3.36 (0.97)		3.77 (1.03)	

*p*-value ***< 0.001, *p*-value **< 0.01, *p*-value *< 0.05; M = mean, SD = standard deviation, η² = effect size; sex (1 = male, 2 = female), age (years: 1 = 45–54, 2 = 55–64, 3 = 65–74, 4 = > 75), partnership (0 = no, 1 = yes), education (1 = school-leaving qualification after 9 years, 2 = school-leaving qualification after 10 years, 3 = technical college certificate or higher education entrance qualification, 4 = other or none), employment (1 = full-time, 2 = part-time, 3 = irregular, 4 = retired, 5 = unemployed)

**Table 2:** Correlations of SPWB mean scores and health, psychosocial variables, and equivalence income (*N* = 3,374)

	SPWB total score	Autonomy	Environmental mastery	Personal growth	Positive relations to others	Purpose in life	Self- acceptance
Depression PHQ-9	− 0.42	− 0.12	− 0.49	− 0.17	− 0.26	− 0.12	− 0.41
Anxiety GAD-7	− 0.34	− 0.11	− 0.45	− 0.12	− 0.19	− 0.03	− 0.35
Sleep JSS-4	− 0.14	− 0.01 n.s.	− 0.21	− 0.07	− 0.09	− 0.01 n.s.	− 0.16
Resilient Coping BRCS	0.39	0.13	0.27	0.37	0.21	0.19	0.29
Social support BS6	0.31	0.07	0.25	0.17	0.29	0.12	0.26
Loneliness	− 0.44	− 0.15	− 0.37	− 0.21	− 0.38	− 0.16	− 0.37
PHQ-stress	− 0.42	− 0.11	− 0.40	− 0.10	− 0.17	− 0.24	− 0.01 n.s.
Somatic diseases	− 0.09	0.01 n.s.	− 0.06	− 0.12	− 0.03 n.s.	− 0.12	− 0.03 n.s.
Equivalence income	0.21	0.06	0.12	0.21	0.07	0.19	0.14

All correlation coefficients significant at 0.001*** level, except those n.s. PHQ = Patient Health Questionnaire, GAD-7 = Generalized Anxiety Disorder-7, JSS-4 = Jenkins Sleep Scale-4, BRCS = Brief Resilient Coping Scale, BS6 = Brief Social Support Scale-6

### Psychometric properties

Item wording and statistics at item level are displayed in Table 3. In order to investigate psychometric properties of the SPWB, we analyzed competing factor models (1–6). (1) The 1-factor model (one general factor of psychological well-being) resulted in χ^2^(135 *df*) = 4362.53, *p* <.001, RMSEA = 0.10 (0.10-0.10), CFI = 0.65 and SRMR = 0.09 with standardized item loadings ranging from 0.16 to 0.73. (2) Showing better model fit, approximating an acceptable one, was observed for the CFA at item level for the correlated 6-factor model with χ^2^(120 *df*) = 2,809.34, *p* <.001, RMSEA = 0.08 (0.08-0.09), CFI = 0.78 and SRMR = 0.08 with standardized item loadings ranging from 0.07 to 0.84. (3) The same applied for the second-order model (six subscales in first order and the general factor in second order) with χ^2^(129 *df*) = 2,927.25, *p* <.001, RMSEA = 0.08 (0.08-0.09), CFI = 0.77 and SRMR = 0.08 with standardized item loadings ranging from 0.08 to 0.82. The corresponding bi-factor models addressing the positively and negatively formulated items lead to improved model fit. (4) The bi-factor model based on the 1-factor model resulted in a good model fit χ^2^(117 *df*) = 1,353.86, *p* <.001, RMSEA = 0.06 (0.06-0.06), CFI = 0.90 and SRMR = 0.05 with standardized item loadings ranging from 0.04 to 0.82. (5) An excellent model fit was found for the bi-factor model based on the correlated 6-factor model with χ^2^(102 *df*) = 625.74, *p* <.001, RMSEA = 0.04 (0.04-0.04), CFI = 0.96 and SRMR = 0.04 with standardized item loadings ranging from 0.03 to 0.78. (6) The bi-factor model based on the second-order model did not converge.

The mix of positively and negatively worded items in the SPWB create homogeneity problems leading to partially problematic statistics. This is especially true for the subscale *Purpose in life* and *Autonomy*. The internal consistency in terms of Cronbach’s alpha are the lowest for both subscales with 0.17 and 0.35. The other subscales reached acceptable values considering the shortness of the subscales (0.42-0.66). A good reliability was observed for the overall scale, with an internal consistency of a = 0.75. Tables 3 and 4 present scale statistics (mean scores, standard deviation, internal consistency) and inter-scale correlations. The current sample of aging adults reported the highest scores on the subscales *Environmental mastery* and *Self-acceptance*, while the lowest score was reported *Autonomy*. Internal consistency of the SPWB scales is acceptable to good except for the subscale *Purpose in Life*. The overall SPWB scale score correlated with the subscale *Environmental mastery* the highest (*r* =.78) and with the subscales *Autonomy* and *Purpose in life* (*r* =.52) the lowest. Inter- scale correlation of the SPWB subscales were low to moderate, *r* =.08 to *r* =.53. The subscales *Environmental mastery* and *Self-acceptance* (*r* =.53) showed the highest correlations, while the subscales *Purpose in life* and *Autonomy* showed the weakest associations (*r* =.08).

**Table 3:** English and German item wording and item statistics of SPWB

Item	Subscale	Item English	Item German	M (SD)
1 (r)	Autonomy	I tend to be influenced by people with strong opinions.	Ich lasse mich leicht beeinflussen von Leuten, die von ihrer Meinung fest überzeugt sind.	3.60 (1.29)
2	Autonomy	I have confidence in my opinions, even if they are contrary to the general consensus.	Ich bin von meiner Meinung überzeugt, auch wenn sie im Widerspruch steht zu dem, was die Allgemeinheit denkt.	3.01 (1.38)
3	Autonomy	I judge myself by what I think is important, not by the values of what others think is important.	Bei der Einschätzung meiner eigenen Person zählt nicht der Wertmaßstab anderer, sondern allein das, was in meinen Augen wichtig ist.	3.22 (1.42)
4	Environmental mastery	In general, I feel I am in charge of the situation in which I live.	Im Großen und Ganzen habe ich das Gefühl, dass ich mein Leben recht gut im Griff habe.	3.96 (1.15)
5 (r)	Environmental mastery	The demands of everyday life often get me down.	Oft erdrückt mich der Alltag mit seinen Anforderungen.	3.59 (1.40)
6	Environmental mastery	I am quite good at managing the many responsibilities of my daily life.	Ich erledige meine vielen alltäglichen Aufgaben und Pflichten ganz gut.	3.91 (1.14)
7	Personal growth	I think it is important to have new experiences that challenge how you think about yourself and the world.	Ich denke es ist wichtig, immer wieder neue Erfahrungen machen, die in Frage stellen, wie man über sich und die Welt nachdenkt.	3.85 (1.17)
8	Personal growth	For me, life has been a continuous process of learning, changing, and growth.	Für mich ist das Leben ein ständiger Lern- und Entwicklungsprozess.	4.06 (1.10)
9 (r)	Personal growth	I gave up trying to make big improvements or changes in my life a long time ago.	Ich habe es schon lange aufgegeben, mein Leben wesentlich verändern oder verbessern zu wollen.	3.47 (1.55)
10 (r)	Positive relations with others	Maintaining close relationships has been difficult and frustrating for me.	Es ist schwierig und anstrengend für mich, enge Beziehungen zu anderen aufrechtzuerhalten.	3.59 (1.46)
11	Positive relations with others	People would describe me as a giving person, willing to share my time with others.	Man könnte mich wohl als einen großzügigen Menschen bezeichnen, der sich Zeit für andere nimmt.	3.46 (1.23)
12 (r)	Positive relations with others	I have not experienced many warm and trusting relationships with others.	Ich habe bisher nur wenige vertrauensvolle und enge Beziehungen erlebt.	3.02 (1.71)
13 (r)	Purpose in life	I live life one day at a time and don’t really think about the future.	Ich hake jeden Tag einzeln ab und mache mir über die Zukunft weiter keine Gedanken.	3.11 (1.57)
14	Purpose in life	Some people wander aimlessly through life, but I am not one of them.	Manche Leute gehen plan- und ziellos durchs Leben, aber zu denen gehöre ich nicht.	3.71 (1.44)
15 (r)	Purpose in life	I sometimes feel as if I’ve done all there is to do in life.	Manchmal fühle ich mich, als ob ich schon alles getan hätte, was es in Leben zu tun gibt.	3.67 (1.46)
16	Self-acceptance	I like most aspects of my personality.	Eigentlich mag ich mich so, wie ich bin.	3.81 (1.18)
17 (r)	Self-acceptance	In many ways, I feel disappointed about my achievements in life.	Irgendwie bin ich mit dem, was ich im Leben erreicht habe, nicht zufrieden.	3.79 (1.40)
18	Self-acceptance	When I look at the story of my life, I am pleased with how things have turned out.	Im Großen und Ganzen bin ich auf mich und mein Leben recht stolz.	3.78 (1.24)

English version by Ryff, Keyes [5]; German version by Staudinger et al. [22]

**Table 4:** Correlations of SPWB mean scores and internal consistency of the scales (*N* = 3,374)

Number of items (reversed)	α	M (SD)		1.	2.	3.	4.	5.	6.
18	0.75	3.58 (0.60)	1. SPWB total						
3 (1)	0.35*	3.27 (0.91)	2. Autonomy	0.52					
3 (1)	0.60	3.82 (0.91)	3. Environmental mastery	0.78	0.31				
3 (1)	0.56	3.77 (1.02)	4. Personal growth	0.68	0.24	0.39			
3 (2)	0.42	3.36 (1.02)	5. Positive relations to others	0.62	0.11	0.32	0.29		
3 (2)	0.17**	3.47 (0.94)	6. Purpose in life	0.52	0.08	0.17	0.32	0.23	
3 (1)	0.66	3.80 (0.99)	7. Self-acceptance	0.71	0.28	0.53	0.33	0.34	0.16

* if (recoded) reversed item excluded, Cronbach’s α would increase to 0.58, ** if positive item excluded, Cronbach’s α would increase to 0.35

## Discussion

In view of the demographic change with increasingly higher life expectancy, healthy aging has become an important research field. Ample evidence supports the crucial role of well-being for physical and mental health, as postulated by positive psychology [36]. In the context of the WHO Global Strategy and Action Plan on Aging and Health, focusing on individuals’ intrinsic capacity and functional ability despite age-related burdens, the search for modifiable protective factors and for measures of healthy aging represents a promising approach to tackle new societal challenges of health care [8]. As Ryff’s SPWB is one of the most well-established multi-dimensional scale for the assessment of psychological well-being, the current study aimed to analyze psychometric property and validity aspects of its German 18-item version.

While in the U.S. test development sample by Ryff, Keyes [5] of adults 25 + years old, *Personal Growth* and *Autonomy* displayed the highest scores, the highest scores in the analyzed German sample aged 45 + years were reported for *Environmental mastery* and *Self-acceptance*. In line with their findings [5], when comparing young vs. middle (30–64 years) vs. older adults (65 + years), participants 65 + years showed the highest scores on these two dimensions. Declining *Personal Growth* and *Purpose in Life* with rising age were likewise observed in our sample. Again in congruence to [5] and other studies analyzing SPWB in aging populations from other countries, no gender differences were established, except for *Positive relations to others* with higher scores in women [12, 37].

Analyses of further sociodemographic variables and SPWB revealed significant results with low effect sizes. Within our sample, all facets of SPWB except for *Autonomy* were lowest for the oldest age range of 75–85 years. The highest scores were found in the age range of 65–74 years, particularly regarding *Autonomy*,* Environmental mastery* and *Self-acceptance*. These findings correspond to Wahl et al. [38] and Henning et al. [39], who found considerable mental health and cognitive gains among the currently young old group compared to previous cohorts, and it may reflect the growing retirement satisfaction, particularly among German white collar workers.

In line with other studies on socioeconomic factors and SPWB [9, 36], we found the strongest effect for education on general psychological well-being, specifically regarding *Personal growth*. Also, individuals with partner generally reported higher scores on SPWB and its facets. Surprisingly, employment status and equivalence income were only weakly associated with general psychological well-being. The fact that almost half of our sample included retired individuals with secured income and without official working status may have led to these results. Retired people turned out to report the highest sense of *Environmental mastery*. In terms of healthy aging, future studies should address this effect to better understand resilient mechanisms among pensioners.

In the next step, we assessed different aspects of validity of the SPWB. First, we investigated its construct validity by analyzing correlations of SPWB with outcomes regarding psychosocial variables, mental and physical health. In sum, SPWB in aging adults is linked with positive health variables. Moderate negative correlations with loneliness, depressiveness, stress, anxiety, and sleep disorder and somatic diseases and positive correlations with resilient coping and social support attested to the validity of the total scale. More specific correlation patterns were found for the subscales: *Environmental mastery* and *Self-acceptance* were most strongly negatively associated with distress, *Positive relations to others* with loneliness, and *Personal growth* with resilient coping. The cumulative index of somatic diseases revealed negative, albeit weak, associations with general well-being. These results complement current studies on aging and age-related diseases suggesting psychological well-being as modifiable risk or protective factor for healthy aging [40]. For instance, psychological well-being assessed with the SPWB turned out to be linked with age-related diseases referring to sensory, cognitive function and neuronal health [41] or inflammation [12, 37].

In line with Ryff, Keyes [5] and a recent analysis of a Swedish 18-item version of SPWB [42], the psychometric properties of the German 18-item version render support for multidimensional models of psychological well-being in our sample of 45–85 years old adults. The observed model fit for the correlated 6-factor model and the second-order factor model with a general factor for psychological well-being with six first-order factors were comparable to previous studies on the factorial validity of the SPWB [19, 20, 40]. However, none of the tested models reached a good model fit. These results are similar to the findings of other studies testing the factorial validity of short forms of SPWB [21, 42]. However, when applying bi-factor models in order to capture the methods effect resulting from positively and negatively formulated items, the model fit improved for the tested models resulting in good (1-factor model) to excellent model fit (correlated 6-factor model). Thus, bi-factor models that take the method effects of positively and negatively formulated items into account are highly recommended for the German SPWB 18-item version.

Analyses of internal consistency of the subscales were comparable to previous studies and reasonable considering the use of ultra-short subscales with only three items [5]. From a statistical point of view, the scale *Purpose in life* did not perform well at all in our analyses. Similarly poor results were found by psychometric papers on SPWB short versions, e.g., with a student sample [21] and within an adult Swedish sample [42]. Hence, results regarding this subscale should be interpreted with caution. Inter-scale correlations of the SPWB subscales were low to moderate, *r* =.08 to *r* =.53. Compared to inter-scale correlations of the original English version [5], the subscales *Environmental mastery* and *Self-acceptance* also showed the highest correlation while correlations of *Autonomy* and *Positive Relations to others* were the lowest. Evaluation of correlation patterns of the SPWB with external criteria support its convergent and discriminant validity.

### Strengths and limitations

The current study is the first analyzing the psychometric properties of the German SPWB 18-item version using a population-based community sample in Germany. Our results provided evidence for the link of general psychological well-being and its different facets with health-related variables paving future epidemiological resilience research in aging adults. Since the main aim was to evaluate the relevance of Ryff’s SPWB for healthy aging, the age range analyzed in this study was fairly homogeneous. Measurement invariance of the German SPWB 18-item version for different groups analyzed should be scrutinized in further investigations. We analyzed cross-sectional data from a large cohort so that no causal inferences can be made. Thus, future studies with the SPWB and health variables of the GHS will target more differentiated multivariate analyses with a longitudinal study design. In addition, a broader age range should be analyzed to gain a better understanding of life trajectories.

## Conclusion

Overall, the German SPWB 18-item version showed to be a valid measure for psychological well-being with room for improvement in terms of reliability. The theoretically proposed multidimensional structure of psychological well-being was mostly supported by our results. Significant associations of psychological well-being and psychosocial variables, mental, respectively physical health strengthen the relevance of psychological well-being as protective factor for healthy aging. Therefore, the German SPWB 18-item version can be used as an indicator for healthy aging in the sense of the WHO definition.

## Data Availability

Written informed consent from GHS study participants does not allow public access to the data. Access to the data in the local database is possible at any time upon request according to the ethics vote. This concept was developed with the local data protection officer and the ethics committee (local ethics committee of the Rhineland-Palatinate Medical Association, Germany). Interested scientists can make their requests to the Gutenberg Health Study Steering Committee (e-mail: info@ghs-mainz.de).

## Abbreviations

SPWB: Scales of Psychological Well-being
GHS: Gutenberg Health Study
WHO: World Health Organization
PHQ-9: Depression module of the Patient Health Questionnaire
GAD-7: Generalized Anxiety Disorder
JSS-4: Jenkins Sleep Survey
BRCS: Brief Resilience Coping Scale
BS6: Brief Social Support Scale
ANOVA: Analysis of Variance
CFS: Confirmatory factor analyses
MLR: Maximum likelihood method
CFI: Comparative fit index
RMSEA: Root mean square error of approximation
SRMR: Standardized root mean square residuals
DF: Degrees of freedom
M: Mean
SD: Standard deviation
